# Inheritance of body size and ultrasound carcass traits in yearling Anatolian buffalo calves

**DOI:** 10.5194/aab-66-325-2023

**Published:** 2023-11-16

**Authors:** Samet Çinkaya, Mustafa Tekerli

**Affiliations:** Department of Animal Science, Faculty of Veterinary Medicine, 03200, Afyonkarahisar, Türkiye

## Abstract

The body size and ultrasound carcass traits are related to the growth and muscling of animals. These characters promise future improvement through genetic selection in animal breeding. In breeding programs, knowing the (co)variance components serves to reveal the performance differences among animals and detection of suitable traits for selection. The research was carried out with 313 Anatolian buffalo calves born in 2019 at 36 farm operations. The least-square means for body weight (BW), wither height (WH), rump height (RH), body length (BL), chest width (CW), hip width (HW), chest circumference (CC), cannon-bone circumference (CBC), longissimus muscle area (LMA), longissimus muscle depth (LMD), and subcutaneous fat thickness (SFT) in yearling calves were 175.41 ± 2.06 kg, 108.35 ± 0.34, 111.85 ± 0.37, 103.74 ± 0.41, 33.93 ± 0.23, 30.56 ± 0.23, 135.18 ± 0.60, 15.69 ± 0.08 cm, 19.36 ± 0.45 cm ${}^{2}$ , 3.086 ± 0.028, and 0.655 ± 0.006 cm, respectively. The direct heritabilities for BW, WH, RH, BL, CW, HW, CC, CBC, LMA, LMD, and SFT were 0.334 ± 0.032, 0.483 ± 0.044, 0.473 ± 0.043, 0.441 ± 0.041, 0.364 ± 0.034, 0.432 ± 0.040, 0.435 ± 0.040, 0.226 ± 0.021, 0.0001 ± 0.000, 0.300 ± 0.026, and 0.539 ± 0.046, respectively. The genetic and phenotypic correlations predicted in this study ranged from 0.02 to 0.90. All the genetic and phenotypic correlations among body size and ultrasound carcass traits were significant ( P<0.01 ), except for the genetic correlation between CW and HW. Some polymorphisms in *PLAG1*, *NCAPG*, *LCORL*, and *HMGA2* genes were analyzed. Two single-nucleotide polymorphisms (SNPs) for *PLAG1* and *NCAPG* genes were found to be monomorphic in this buffalo population. Meanwhile, the effects of two SNPs in the *LCORL* and *HMGA2* genes were not significant but showed some tendencies in the aspects of least-square means. The results of the study indicated that the Anatolian buffaloes have the potential to improve in growth and muscling characteristics.

## 1 Introduction

The buffalo is a robust and thrifty animal. It is one of the species that people benefit from. e.g., meat, milk, leather, and manure production, and they are used as draw animal in some distinct regions of the world. When compared, water buffalo have fallen short cattle in the aspect of production, but it is still an alternative production resource throughout the world (İzmen and Spöttel, 1937; Uslu, 1970; Şahin et al., 2013; Koçak et al., 2019). *Bubalus*, known as water buffalo, is in the same subfamily with *Bos*, *Syncerus*, and *Bison*. The order of water buffalo in taxonomy is as follows: Mammalia class, Artiodactyla order, Bovidae family, Bovinae subfamily, *Bubalus* genus, and *Bubalus bubalis* species. Domestic buffaloes are divided into two subspecies. These are riverine (*Bubalus bubalis bubalis*, 2n=50 chromosomes) and swamp (*Bubalus bubalis kerabau*, 2n=48 chromosomes) buffaloes (Soysal, 2009; ITIS, https://itis.gov, last access: 14 November 2022).

Studies about body measurements are generally conducted on adult buffaloes (Sindhu and Pal, 1980; Peeva, 1991; Ahmad et al., 2013; Dhillod et al., 2017; de Melo et al., 2018; Riaz et al., 2018; Korejo et al., 2019; Dahiya et al., 2020; Mirza et al., 2020). However, research focusing on yearling buffaloes are scarce (Gürcan et al., 2011; Gavit et al., 2013; Çelikeloğlu et al., 2019; Ağyar et al., 2022). Some scientists (Thevamanoharan et al., 2001; Mirza et al., 2020) were interested in genetic parameters of lactating buffaloes except for Vankov and Peeva (1994). The genetic background and relationships of body size traits have been relatively uncertain in different breeds even today.

Ultrasound carcass traits in adult buffaloes were measured and reported by Harada et al. (1997), Jorge et al. (2007), Rebak et al. (2010), and Singh et al. (2018). Taborda et al. (2015) notified the community that the heritabilities for longissimus muscle area (LMA) and rump fat thickness were 0.256 and 0.214.

The number of studies aiming to understand the genetic basis of growth and development in humans and cattle showed a significant increase in the last decades. Single-nucleotide polymorphisms (SNPs) in *PLAG1, NCAPG, LCORL*, and *HMGA2* genes were found to be associated with height in humans (Gudbjartsson et al., 2008). Pryce et al. (2011) also reported that the same genes had an effect on RH in cattle by similar functionalities.

The objectives of this study were to reveal the hereditary background of body size and ultrasound carcass traits and enlighten some SNPs in *PLAG1, NCAPG, LCORL*, and *HMGA2* genes in yearling Anatolian buffaloes.

## 2 Materials and methods

This study was reviewed and approved by the experimental animal ethics committee of Afyon Kocatepe University (AKUHADYEK, 49533702/258 and 210/20).

The data consisted of 2165 body measurements and 730 ultrasound carcass trait records from a total of 313 yearling Anatolian buffalo registered to the “Community Based Buffalo Improvement Project” governed by the General Directorate of Agricultural Research and Policies of the Ministry of Agriculture and Forestry. All measurements were taken from February to August 2019 in 36 farm operations.

Body size traits of an animal with a normal posture standing on a weighing scale were measured using measuring stick, caliper, and tape as described by Batu (1951), Kendir (1960), Arpacık (1982), and Gilbert et al. (1993). Body measurements were wither height (the highest point on the withers), rump height (the highest point on the rump), body length (the distance between *caput humerii* and *tuber ischiadicum*), chest width (the distance between two *caput humerii*), hip width (the distance between two Tuber coxae), chest circumference (circumference immediately posterior of the front legs), and cannon-bone circumference (the circumference of the left metacarpus at its narrowest).

**Figure 1:**
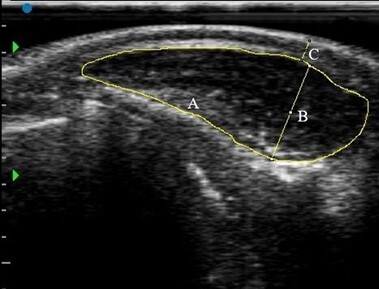
The real-time ultrasound image and its interpretation in a buffalo calf (borders A and depth B of longissimus muscle, and subcutaneous fat thickness (SFT) labeled C).

After the body weight and measurements were recorded, the region between the 13th dorsal vertebra and the first lumbar vertebra (Hwang et al., 2014) on the left side of the animals was scanned for the ultrasound measurements. The hair of the measurement area was clipped, and then all the hairs were removed with the help of a razor blade to obtain a good-quality image. The conductive medium was ultrasound gel. The SIUI CTS-800 scanner with linear and back-fat probes was used for imaging. An instant ultrasound image was obtained for each animal. The images were processed by ImageJ (Schneider et al., 2012) software after the calibration of pixels (Fig. 1).

The values at 12 months of age for all traits were estimated from successive measurements by interpolation or extrapolation as described by Gürtan (1979) using an in-house add-in running in Microsoft Excel.

Genomic DNA was extracted from blood samples using a modified method described by Boom et al. (1990). We selected SNPs coded as rs109231213 (Karim et al., 2011) and c.132T > G (Setoguchi et al., 2009) for *PLAG1* and *NCAPG* genes. These SNPs were examined for nucleotide variation by the Sanger sequence (ABI 3500 genetic analyzer) using pool DNA (12 animals for each). AX-85166825 and AX-85179490 coded SNPs located in the upstream and downstream regions of *LCORL* and *HMGA2* genes were preferred. The PCR-RFLP (restriction fragment length polymorphism) method was applied for the detection of variants. For this purpose, BccI and BmrI restriction enzymes were used. Genotyping was completed by agarose gel electrophoresis and imaging (Bio-Vision, Vilber Lourmat).

**Table 1:** Least-square means for body size traits in Anatolian buffalo.

Factors		n	BW, kg	$n^{1}$	WH, cm	RH, cm	BL, cm	CW, cm	HW, cm	CC, cm	CBC, cm
	μ	261	175.41 ± 2.06	272	108.35 ± 0.34	111.85 ± 0.37	103.74 ± 0.41	33.93 ± 0.23	30.56 ± 0.23	135.18 ± 0.60	15.69 ± 0.08
Birth season								${}^{***}$	${}^{***}$		
	Spring	152	177.13 ± 2.66	150	108.39 ± 0.44	111.36 ± 0.48	103.70 ± 0.52	35.43 ± 0.30 ${}^{a}$	29.03 ± 0.30 ${}^{b}$	135.43 ± 0.78	15.74 ± 0.10
	Summer	109	173.70 ± 3.08	122	108.31 ± 0.49	112.33 ± 0.53	103.77 ± 0.59	32.43 ± 0.33 ${}^{b}$	32.08 ± 0.33 ${}^{a}$	134.93 ± 0.86	15.64 ± 0.11
Sex			${}^{***}$		${}^{*}$		${}^{***}$		${}^{***}$		${}^{***}$
	Male	150	182.83 ± 2.62 ${}^{a}$	150	109.00 ± 0.43 ${}^{a}$	112.46 ± 0.47	104.84 ± 0.51 ${}^{a}$	34.09 ± 0.29	31.50 ± 0.29 ${}^{a}$	136.07 ± 0.76	16.02 ± 0.10 ${}^{a}$
	Female	111	168.00 ± 2.96 ${}^{b}$	122	107.70 ± 0.48 ${}^{b}$	111.24 ± 0.53	102.64 ± 0.58 ${}^{b}$	33.77 ± 0.33	29.62 ± 0.33 ${}^{b}$	134.30 ± 0.85	15.36 ± 0.11 ${}^{b}$
Dam age											
	≤6 year	94	175.06 ± 3.13	103	108.44 ± 0.50	112.09 ± 0.55	104.04 ± 0.60	33.63 ± 0.34	30.77 ± 0.34	135.40 ± 0.88	15.65 ± 0.11
	> 6 year	167	175.77 ± 2.49	169	108.26 ± 0.42	111.60 ± 0.45	103.44 ± 0.50	34.23 ± 0.28	30.35 ± 0.28	134.97 ± 0.73	15.72 ± 0.09
Birth weight										${}^{*}$	
	< 33 kg	135	171.40 ± 2.88	148	107.68 ± 0.47	111.11 ± 0.51	103.21 ± 0.56	33.49 ± 0.32	30.74 ± 0.32	133.76 ± 0.83 ${}^{b}$	15.58 ± 0.11
	≥33 kg	126	179.43 ± 3.31	124	109.02 ± 0.54	112.58 ± 0.59	104.27 ± 0.64	34.37 ± 0.36	30.37 ± 0.37	136.61 ± 0.95 ${}^{a}$	15.80 ± 0.12

Abbreviations: BW, body weight; WH, wither height; RH, rump height; BL, body length; CW, chest width; HW, hip width; CC, chest circumference; CBC, cannon-bone circumference. ${}^{1}$ The number of animals used in the analysis for WH, RH, BL, CW, HW, CC, and CBC. ${}^{*}$ P<0.05 ; ${}^{**}$ P<0.01 ; ${}^{***}$ P<0.001 . ${}^{a,b}$ Means with different superscripts within a variable differ significantly ( P<0.05 ).

**Table 2:** Least-square means for ultrasound carcass traits in Anatolian buffalo.

Factors		n	LMA, cm ${}^{2}$	n	LMD, cm	n	SFT, cm
μ		102	19.36 ± 0.45	309	3.086 ± 0.028	309	0.655 ± 0.006
Birth season							
	Spring	43	19.25 ± 0.69	176	3.121 ± 0.036	176	0.647 ± 0.008
	Summer	59	19.48 ± 0.63	133	3.052 ± 0.041	133	0.663 ± 0.009
Sex					*		
	Male	48	19.49 ± 0.62	168	3.149 ± 0.037 ${}^{a}$	168	0.647 ± 0.008
	Female	54	19.24 ± 0.64	141	3.024 ± 0.040 ${}^{b}$	141	0.664 ± 0.009
Dam age							
	≤6 year	38	18.90 ± 0.71	118	3.065 ± 0.042	118	0.651 ± 0.009
	> 6 year	64	19.83 ± 0.54	191	3.108 ± 0.035	191	0.660 ± 0.008

Abbreviations: LMA, longissimus muscle area; LMD, longissimus muscle depth; SFT, subcutaneous fat thickness. ${}^{*}$ P<0.05 .

The effects of farm, birth season, sex, dam age and birth weight on body measurements and ultrasound carcass traits (except for birth weight) were analyzed by the method of least squares using the following model:

1 $$Y_{ijklmn}=\mu +F_{i}+{\mathrm{BS}}_{j}+S_{k}+{\mathrm{DA}}_{l}+{\mathrm{BW}}_{m}+e_{ijklmn},$$

where $Y_{ijklmn}=n$ th observation in the m th birth weight, l th dam age, k th sex, j th birth season, and i th farm; μ = the overall mean; $F_{i}=$ the effect of i th farm ( i = 1, 2, …, 35, and 36); BS ${}_{j}=$ the effect of j th birth season ( j = spring, summer); $S_{k}=$ the effect of k th sex ( k = male, female); DA ${}_{l}=$ the effect of l th dam age ( l =≤6 , > 6 year); BW ${}_{m}=$ the effect of m th birth weight ( m = <33 , ≥ 33 kg); $e_{ijklmn}=$ random error N (0, $\sigma^{2}$ ).

**Table 3:** Estimation of variance components for body size and ultrasound carcass traits in Anatolian buffalo.

		Parameters						
Traits	n	$\sigma_{a}^{2}$	$\sigma_{m}^{2}$	$\sigma_{e}^{2}$	$\sigma_{P}^{2}$	$h_{a}^{2}$	$h_{m}^{2}$	$h_{T}^{2}$
BW	261	260.172 ± 0.000	503.610 ± 244.646	16.163 ± 221.516	779.945 ± 75.420	0.334 ± 0.032	0.646 ± 0.291	0.656 ± 0.140
WH	272	10.391 ± 0.000	0.002 ± 7.651	11.139 ± 7.659	21.534 ± 1.978	0.483 ± 0.044	0.000 ± 0.355	0.483 ± 0.178
RH		12.321 ± 0.000	0.923 ± 9.146	12.812 ± 9.120	26.056 ± 2.391	0.473 ± 0.043	0.035 ± 0.351	0.491 ± 0.175
BL		13.746 ± 0.000	9.111 ± 10.730	8.296 ± 10.341	31.153 ± 2.887	0.441 ± 0.041	0.292 ± 0.338	0.587 ± 0.166
CW		3.612 ± 0.000	2.337 ± 3.533	3.982 ± 3.441	9.931 ± 0.918	0.364 ± 0.034	0.235 ± 0.351	0.481 ± 0.173
HW		4.536 ± 0.000	2.685 ± 3.745	3.275 ± 3.629	10.498 ± 0.974	0.432 ± 0.040	0.256 ± 0.352	0.560 ± 0.173
CC		29.698 ± 0.000	28.433 ± 22.935	10.173 ± 21.697	68.305 ± 6.359	0.435 ± 0.040	0.416 ± 0.325	0.643 ± 0.159
CBC		0.248 ± 0.000	0.837 ± 0.332	0.014 ± 0.297	1.099 ± 0.103	0.226 ± 0.021	0.761 ± 0.276	0.606 ± 0.133
LMA	104	0.016 ± 0.000	0.010 ± 10.761	15.291 ± 10.484	15.294 ± 2.366	0.0001 ± 0.000	0.000 ± 0.704	0.001 ± 0.351
LMD	313	0.053 ± 0.000	0.088 ± 0.060	0.036 ± 0.056	0.178 ± 0.015	0.300 ± 0.026	0.496 ± 0.326	0.547 ± 0.159
SFT	313	0.004 ± 0.000	0.002 ± 0.002	0.001 ± 0.002	0.008 ± 0.000	0.539 ± 0.046	0.315 ± 0.277	0.697 ± 0.137

Abbreviations: BW, body weight; WH, wither height; RH, rump height; BL, body length; CW, chest width; HW, hip width; CC, chest circumference; CBC, cannon-bone circumference; LMA, longissimus muscle area; LMD, longissimus muscle depth; SFT, subcutaneous fat thickness; $\sigma_{a}^{2}$ , direct additive genetic variance; $\sigma_{m}^{2}$ , maternal genetic variance; $\sigma_{e}^{2}$ , residual variance; $\sigma_{P}^{2}$ , phenotypic variance; $h_{a}^{2}$ , direct additive heritability; $h_{m}^{2}$ , maternal heritability; $h_{T}^{2}$ , total heritability.

The farms with at least four animals for all traits were used in the analyses. The birth season consisted of spring and summer subgroups. Sex and dam age were divided into two groups (male and female, ≤ 6 and > 6 years of age). The least-square analyses were applied by Minitab 18, and the Tukey test was used for multiple comparisons.

Estimation of (co)variance components for body measurements and ultrasound carcass traits were obtained by restricted maximum likelihood (REML) using WOMBAT (Meyer, 2007) software, considering univariate animal model and numerator relationship matrix to obtain a more accurate breeding value (Meyer, 1992; Çinkaya et al., 2019). The model used for the analyses was as follows:

$$Y=X\beta +Z_{a}a+Z_{m}m+e\mathrm{Cov}\left(a,m\right)=0,$$

where Y is the vector of observations, β is the vector of nongenetic significant fixed effects, a is the vector of random additive genetic effects, m is the vector of random maternal genetic effects, and e is the unknown random vector of residuals. X , $Z_{a}$ , and $Z_{m}$ are incidence matrices associating observations to fixed, additive genetic, and maternal genetic effects, respectively.

The genetic correlations for body measurements and ultrasound carcass traits were calculated from the estimated breeding values (EBVs) of related traits with the following formula (Calo et al., 1973; Falconer and Mackay, 1996; Mitchell et al., 2005);

$$r_{G}(XY)=\frac{{\mathrm{Cov}}_{XY}}{\sqrt{\left(\sigma_{aX}^{2}\cdot \sigma_{aY}^{2}\right)}},$$

where $r_{G}(XY)$ is the genetic correlation between X and Y traits, ${\mathrm{Cov}}_{XY}$ is the covariance between EBVs for X and Y , $\sigma_{aX}^{2}$ is the direct additive genetic variance of trait X , and $\sigma_{aY}^{2}$ is the direct additive genetic variance of trait Y .

**Table 4:** Genetic (upper diagonal) and phenotypic (lower diagonal) correlations among body size and ultrasound carcass traits of Anatolian buffalo.

Traits	BW	WH	RH	BL	CW	HW	CC	CBC	LMA	LMD	SFT
BW	–	0.78 ${}^{**}$	0.79 ${}^{**}$	0.82 ${}^{**}$	0.69 ${}^{**}$	0.62 ${}^{**}$	0.88 ${}^{**}$	0.76 ${}^{**}$	0.71 ${}^{**}$	0.75 ${}^{**}$	0.65 ${}^{**}$
WH	0.73 ${}^{**}$	–	0.90 ${}^{**}$	0.75 ${}^{**}$	0.51 ${}^{**}$	0.54 ${}^{**}$	0.76 ${}^{**}$	0.63 ${}^{**}$	0.51 ${}^{**}$	0.56 ${}^{**}$	0.50 ${}^{**}$
RH	0.77 ${}^{**}$	0.90 ${}^{**}$	–	0.75 ${}^{**}$	0.53 ${}^{**}$	0.56 ${}^{**}$	0.78 ${}^{**}$	0.67 ${}^{**}$	0.53 ${}^{**}$	0.59 ${}^{**}$	0.52 ${}^{**}$
BL	0.77 ${}^{**}$	0.75 ${}^{**}$	0.75 ${}^{**}$	–	0.51 ${}^{**}$	0.57 ${}^{**}$	0.72 ${}^{**}$	0.67 ${}^{**}$	0.62 ${}^{**}$	0.64 ${}^{**}$	0.54 ${}^{**}$
CW	0.69 ${}^{**}$	0.51 ${}^{**}$	0.53 ${}^{**}$	0.51 ${}^{**}$	–	0.02	0.63 ${}^{**}$	0.55 ${}^{**}$	0.38 ${}^{**}$	0.57 ${}^{**}$	0.46 ${}^{**}$
HW	0.56 ${}^{**}$	0.53 ${}^{**}$	0.56 ${}^{**}$	0.57 ${}^{**}$	0.02 ${}^{**}$	–	0.60 ${}^{**}$	0.60 ${}^{**}$	0.46 ${}^{**}$	0.45 ${}^{**}$	0.49 ${}^{**}$
CC	0.85 ${}^{**}$	0.77 ${}^{**}$	0.79 ${}^{**}$	0.72 ${}^{**}$	0.64 ${}^{**}$	0.60 ${}^{**}$	–	0.74 ${}^{**}$	0.65 ${}^{**}$	0.67 ${}^{**}$	0.59 ${}^{**}$
CBC	0.76 ${}^{**}$	0.63 ${}^{**}$	0.67 ${}^{**}$	0.67 ${}^{**}$	0.56 ${}^{**}$	0.60 ${}^{**}$	0.76 ${}^{**}$	–	0.68 ${}^{**}$	0.67 ${}^{**}$	0.61 ${}^{**}$
LMA	0.67**	0.49 ${}^{**}$	0.52 ${}^{**}$	0.61 ${}^{**}$	0.35 ${}^{**}$	0.46 ${}^{**}$	0.64 ${}^{**}$	0.68 ${}^{**}$	–	0.82 ${}^{**}$	0.56 ${}^{**}$
LMD	0.73 ${}^{**}$	0.56 ${}^{**}$	0.59 ${}^{**}$	0.65 ${}^{**}$	0.56 ${}^{**}$	0.46 ${}^{**}$	0.67 ${}^{**}$	0.67 ${}^{**}$	0.83 ${}^{**}$	–	0.53 ${}^{**}$
SFT	0.62 ${}^{**}$	0.50 ${}^{**}$	0.52 ${}^{**}$	0.53 ${}^{**}$	0.46 ${}^{**}$	0.48 ${}^{**}$	0.60 ${}^{**}$	0.62 ${}^{**}$	0.54 ${}^{**}$	0.53 ${}^{**}$	–

Abbreviations: BW, body weight; WH, wither height; RH, rump height; BL, body length; CW, chest width; HW, hip width; CC, chest circumference; CBC, cannon-bone circumference; LMA, longissimus muscle area; LMD, longissimus muscle depth; SFT, subcutaneous fat thickness. ${}^{**}$ P<0.01 .

**Table 5:** Direct (diagonal) and correlated responses of selection for body size and ultrasound carcass traits in Anatolian buffalo.

	Responses										
Auxiliary trait	BW	WH	RH	BL	CW	HW	CC	CBC	LMA	LMD	SFT
BW	9.3278 ${}^{a}$	0.6486	0.6638	0.7136	0.6610	0.5452	0.7711	0.9239	41.0328	0.7914	0.5117
WH	0.9380	2.2413 ${}^{b}$	0.9095	0.7849	0.5875	0.5710	0.7600	0.9210	35.4441	0.7106	0.4733
RH	0.9401	0.8906	2.4144 ${}^{b}$	0.7767	0.6042	0.5860	0.8134	0.9693	36.4507	0.7408	0.4871
BL	0.9422	0.7166	0.7242	2.4614 ${}^{b}$	0.5614	0.5759	0.7249	0.9359	41.1729	0.7760	0.4884
CW	0.7203	0.4427	0.4649	0.4633	1.1471 ${}^{b}$	0.0184	0.5763	0.6980	22.9263	0.6279	0.3780
HW	0.7051	0.5107	0.5352	0.5642	0.0218	1.3997 ${}^{b}$	0.5979	0.8295	30.2343	0.5400	0.4387
CC	1.0043	0.7212	0.7480	0.7151	0.6887	0.6021	3.5951 ${}^{b}$	1.0266	42.8704	0.8068	0.5300
CBC	0.6252	0.4309	0.4631	0.4796	0.4334	0.4340	0.5334	0.2369 ${}^{b}$	32.3268	0.5815	0.3950
LMA	0.0123	0.0088	0.0077	0.0093	0.0063	0.0070	0.0099	0.0143	0.0004 ${}^{c}$	0.0150	0.0076
LMD	0.7108	0.4413	0.5279	0.5279	0.5175	0.3750	0.5564	0.7719	44.9132	0.1266 ${}^{b}$	0.3954
SFT	0.8257	0.5282	0.5970	0.5970	0.5598	0.5473	0.6568	0.9420	41.1133	0.7104	0.0482 ${}^{b}$

Abbreviations: BW, body weight; WH, wither height; RH, rump height; BL, body length; CW, chest width; HW, hip width; CC, chest circumference; CBC, cannon-bone circumference; LMA, longissimus muscle area; LMD, longissimus muscle depth; SFT, subcutaneous fat thickness. ${}^{a}$ : kg; ${}^{b}$ : cm; ${}^{c}$ : cm ${}^{2}$ .

The phenotypic correlations were calculated as Pearson correlation coefficient with the adjusted data for significant environmental effects. Corrections were performed with an in-house computer software named “Damızlık Asistanı” developed by Mustafa Tekerli̇. Correlations were calculated by Minitab 18.

The expected genetic gains (Venge and Christensen, 1969; Syrstad, 1970; Falconer and Mackay, 1996; Boareki et al., 2020) were predicted by the following equations:

$$\Delta G_{X}=\;i\cdot h_{aX}^{2}\cdot \sigma_{PX},$$

where $\Delta G_{X}$ is the direct response to selection on trait X ; i is the intensity of selection, assumed to be equal to 1; $h_{aX}^{2}$ is the direct additive heritability of trait X ; and $\sigma_{PX}$ is the phenotypic standard deviation of trait X .

Relative correlated responses in the i th trait were calculated with the following formula (Rao and Sundaresan, 1979):

$${\mathrm{CR}}_{i}=r_{G}\left(i,j\right)\cdot \frac{h_{j}}{h_{i}}.$$

Here, ${\mathrm{CR}}_{i}$ is the correlated response of i th trait; $r_{G}\left(i,j\right)$ is the genetic correlation between traits i and j ; $h_{j}$ is the square root of the heritability of j th trait against which selection is directed; and $h_{i}$ is the square root of the heritability of i th trait.

The effects of SNPs were tested with one-way ANOVA (Minitab 18) using the data adjusted for significant environmental effects.

## 3 Results and discussion

The least-square means and ANOVA results for body measurements and ultrasound carcass traits of yearling Anatolian buffaloes are presented in Tables 1–2.

The farm had a significant ( P<0.001 ) effect on all traits. Yılmaz et al. (2017), Erdoğan et al. (2021), Kaplan (2021), and Alkoyak and Öz (2022) reported similar results for the effects of village and rearing site on Anatolian buffaloes. The birth season was found to be significant ( P<0.001 ) in only CW and HW. Sex had a significant ( P<0.05 ) effect on BW, WH, BL, CBC, and LMD. Similar findings were obtained in different studies (Thiruvenkadan et al., 2009; Kul et al., 2017; Çelikeloğlu et al., 2019; Erdoğan et al., 2021; Kaplan, 2021; Alkoyak and Öz, 2022). BW values (175.41 ± 2.06 kg) were found to be higher than the results of earlier research (Thiruvenkadan et al., 2009; Akhtar et al., 2012; Şekerden, 2014; Pandya et al., 2015; Kul et al., 2017; Yılmaz et al., 2017; Erdoğan et al., 2021; Kaplan, 2021) for Murrah, Nili-Ravi, Surti, and Anatolian breeds, whereas higher values were reported by some studies (Shahin et al., 2010; Mendes Malhado et al., 2012; Vergara et al., 2012; Falleiro et al., 2013; Agudelo-Gómez et al., 2015; Shahjahan et al., 2017; Çelikeloğlu et al., 2019; El-den et al., 2020; Medrado et al., 2021). The least-square means for body measurements were similar to the results of literature (Uslu, 1965; Gürcan et al., 2011; Gavit et al., 2013; Çelikeloğlu et al., 2019). No information was encountered in the literature for ultrasound carcass traits of yearling buffaloes. This study was the first investigation in this context. Differences in the ANOVA may be due to age, breed, care, breeding, geography, and statistical models applied.

The variance components and direct additive, maternal, and total heritabilities estimated for the studied traits are shown in Table 3. The direct additive heritability for BW was found to be consonant with Medrado et al. (2021) and lower than the findings of Salces et al. (2006), Mendes Malhado et al. (2007), Shahin et al. (2010), Akhtar et al. (2012), Mendes Malhado et al. (2012), Vergara et al. (2012), Falleiro et al. (2013), Salces et al. (2013), Gupta et al. (2015), Pandya et al. (2015), El-den et al. (2020), and Kaplan (2021).

The estimate of direct additive heritability for WH was higher than that of Vankov and Peeva (1994). The direct heritability of rump height was higher than the findings in Nili-Ravi buffaloes (Mirza et al., 2020). The direct additive heritability estimates for BL were greater than those reported by Vankov and Peeva (1994) for yearlings and Thevamanoharan et al. (2001), and Mirza et al. (2020) for lactating buffaloes. The additive heritability for CC was 0.435 ± 0.040. While Vankov and Peeva (1994) reported a lower estimate for yearlings in this trait, Thevamanoharan et al. (2001) found a higher value in lactating buffaloes.

The estimate of heritability for LMA was lower than the result of Taborda et al. (2015) in buffaloes at 18 months of age. The data may be insufficient to detect a pronounced heritability estimate for LMA in our study. However, the direct additive heritability of SFT (0.539 ± 0.046) was larger according to the report of Taborda et al. (2015). The data frame and the statistical model may have caused differences between studies.

Maternal heritability for BW was found to be 0.646 ± 0.291 and higher than the results revealed by Vergara et al. (2012) and Falleiro et al. (2013) for Colombian and Mediterranean breeds. Buffalo breeders traditionally allow the calves to suckle only one teat of mothers during milking. Some high and significant maternal heritabilities obtained for body measurements and ultrasound carcass traits in our study may be an indicator the genetic capacity of mothers for milk production. Thus, Kushwaha et al. (2008) also emphasized the importance of maternal genetic effects, presumably reflecting differences in milk production.

The genetic and phenotypic correlations among the traits are presented in Table 4. All genetic and phenotypic correlations were significant ( P<0.01 ) and ranged from moderate to high (except for CW and HW). These are the first results in connection with the body measurements and ultrasound carcass traits in the Anatolian buffalo population. No study was found discussing the genetic correlations among body size and ultrasound carcass traits in buffaloes.

The phenotypic correlations between BW and the other measurement traits were moderate to high (0.56–0.85) in the study. Opposite to these results, several authors (Sindhu and Pal, 1980; Paul and Das, 2012; Ahmad et al., 2013; Dhillod et al., 2017) reported positive but low to moderate correlations in different breeds. The correlations except for between CW and HW for body measurements were in the range of the literature (Paul and Das, 2012; Ahmad et al., 2013; Dhillod et al., 2017; de Melo et al., 2018; Nicolas et al., 2018; Dahiya et al., 2020; Ağyar et al., 2022). The significant and moderate genetic and phenotypic correlations between body measurements and ultrasound carcass traits indicated that newly integrated ultrasound techniques could improve the allometric traits. The correlations among ultrasound carcass traits themselves ranged from 0.53 to 0.83. These findings are in tune with the results of Andrighetto et al. (2010) for SFT and LMA. Accordingly, breeders may choose to measure muscle depth because of its practicality.

The direct and correlated responses between studied traits are presented in Table 5. The results showed that the most effective auxiliary trait is CC for improving others.

After sequencing analysis, SNPs investigated for *PLAG1* and *NCAPG* genes were found to be monomorphic. In the *LCORL* gene, two genotypes were obtained. TT and TC genotypes were found in 227 and 9 animals with the means (BW) of 161.12 and 165.89 kg, respectively. CC ( n : 162; $\bar{X}$ : 161.12 kg), TC ( n : 70; $\bar{X}$ : 161.72 kg), and TT ( n : 3; $\bar{X}$ : 166.30 kg) genotypes were detected in the *HMGA2* gene. The differences between genotypes in both genes were not significant.

## 4 Conclusions

This study is the first paper on genetic parameters in Anatolian buffalo for body size and ultrasound carcass traits. The genetic parameters obtained for body size and ultrasound carcass traits have shown that the Anatolian buffaloes could be genetically improved. The highest heritability was calculated in SFT, followed by WH, RH, BL, and CC. Genetic and phenotypic correlations among the traits were found to be in a desirable way and generally moderate to high. CC could be used as a criterion for indirect selection in animal improvement programs. Although there were no significant effects of SNPs in *LCORL* and *HMGA2* genes, calves carrying genotypes of TC in *LCORL* and TT in *HMGA2* had a tendency to be slightly heavier.

## Data Availability

Data will be made available upon reasonable request.
